# Supplementary material for: Content-rich biological network constructed by mining PubMed abstracts
Source: BMC Bioinformatics. 2004 Oct 8;5:147. doi: 10.1186/1471-2105-5-147 (PMC528731; doi:10.1186/1471-2105-5-147)
Supplement: Additional File 2 — The original results of the above study (non-essential files are deleted to keep the file size under the limit set by BMC bioinformatics). [file 1471-2105-5-147-S2.bz2 › chilibotAdditionalFile2/dip10/455ID10733106E10472/html/CCR3_CCL24.html]

 


 **CCR3** and **CCL24** 
  
Found 42 abstracts in PubMed, retrieved 10.  
 

 What does Google say? 
 PDF only 
| .edu only 

---

**Interactive relationship** (e.g. stimulation, inhibition, etc)

**Neutral relationship**- The Eotaxin gene family Eotaxin1, Eotaxin2  [ **CCL24** ]  and Eotaxin3 recruits and activates  **CCR3**  bearing cells such as eosinophils, mast cells and Th2 lymphocytes that play a major role in allergic disorders.  Ref: 12761043 Hum Mol Genet< MedlineTA>Hum Mol Genet,

**Non-interactive relationship** (e.g. studied together, co-existance, homology, etc.)

- Stably transfected Cynomolgus  **CCR3**  bound human eotaxin CCL11 with similar kinetics Kd 240 pM and was responsive to human  **CCR3**  ligands eotaxin CCL11, eotaxin 2  [ **CCL24** ]   **CCL24** , and MCP4 CCL13 in Ca 2 mobilization and chemotaxis assays, thus provides a useful alternative species model system for the analysis of modulators of eotaxin  **CCR3**  induced signaling and activation.  Ref: 12421570 Cytokine, 2002
- In contrast, the  **CCR3**  agonists CCL11,  **CCL24**  and CCL26 are involved in the recruitment of cells such as eosinophils and basophils during Th2 responses.  Ref: 12884299 Eur J Immunol, 2003
- The eotaxin group chemokines eotaxin, eotaxin 2  [ **CCL24** ] , and eotaxin 3 share only 35 41% sequence identity but are all agonists for the receptor  **CCR3** .  Ref: 12486712 Proteins, 2003
- **CCR3**  mediates in vitro chemotactic responses to the known ligands, eotaxin and eotaxin 2  [ **CCL24** ] , because the DC response to these chemokines is inhibited by  **CCR3**  specific mAbs.  Ref: 12218106 J Immunol, 2002
- Like eotaxin and eotaxin 2  [ **CCL24** ] , eotaxin 3 mediates its activity through  **CCR3** .  Ref: 12689946 Blood< MedlineTA>Blood, 2002
